# *Bioengineering & Translational Medicine*: Year 2020 in review

**DOI:** 10.1002/btm2.10178

**Published:** 2020-08-26

**Authors:** Samir Mitragotri

**Affiliations:** ^1^ Harvard University Cambridge Massachusetts USA

2020 marks an important year in the evolution of *BioTM*. We received our first impact factor of 6.091 this year. This is a massive accomplishment for all stakeholders of the journal. The journal was formally launched in 2015 and we published our first issue in 2016. Within a short period of 4 years, we were able to reach this significant milestone.

Huge thanks to all *BioTM* authors who trusted the journal with their precious manuscripts in the early, unproven days. Submission of high‐quality manuscripts is the principal reason that led to an impressive launching impact factor. *BioTM* welcomes manuscripts in all aspects of bioengineering including drug delivery, drug discovery and development, tissue engineering, synthetic biology, biosensors, organoids and organ‐mimetic systems, stem cell therapies, gene therapies, immunotherapies, patient‐targeted therapies, medical devices, regenerative medicine, implant/patient interface, computational modeling, and bioinformatics.

Areas including drug delivery, gene therapy, sirRNA delivery, regenerative medicine, nanomedicine, biomedical devices, and cell therapy have been particularly well represented in the manuscripts recently submitted to *BioTM*. This is expected because these fields represent some of the most active research areas at the interface of medicine and engineering, especially those that focus on developing/testing novel materials or devices, for example, nanoparticles and microneedles for drug delivery. Indeed, some of the recent *BioTM* papers in drug delivery have focused on the use of microneedles for vaccine delivery.^1^, ^2^, ^3^ These papers demonstrated novel microneedle designs, for example, self‐healing microneedles for encapsulation, as well as translational studies to nonhuman primate studies for hepatitis B vaccine. Along the same general theme, studies on ocular drug delivery devices were also featured prominently.^4^, ^5^ The studies described novel designs of protein‐delivery systems as well as studies in nonhuman primates demonstrating long‐lasting release of proteins in the eye. Collectively, the reports along the translational axis of drug delivery devices represent a growing need in the field, and we are delighted to see those represent in our issues. On a more fundamental side, microfluidic devices leading to model systems for blood–brain barrier were also described.^6^ Such devices are playing an increasing role in in vitro optimization and acceleration of drug development timescales.

Tissue engineering was also a commonly discussed subject in recent *BioTM* papers, also consistent with the large community of researchers working at the interface of engineering and regenerative medicine. Covered tissue engineering topics included engineering of hydrogel and decellularized matrices and generation of tissue‐specific blood vessels.^7^, ^8^, ^9^ Polymeric materials of various forms were discussed, including composite materials, hydrogels, and capsules.^10^, ^11^, ^12^ These reports represent the great activity of research focused on understanding the design features of polymeric materials, polymer‐tissue interface, as well as in vivo assessment of their biological functions.

Consistent with general literature trends, a significant number of recent papers were focused on nanomedicine. Topics included translatable mouse models for cancer,^13^ new designs of nanoparticles,^14^, ^15^ and scalable synthesis methods for nanoparticles.^16^, ^17^ Translationally focused reports, for example, novel tumor models for nanomedicine and scalable synthesis methods, represent a particular need of time and we look forward to receiving more similar translationally focused nanomedicine manuscripts in future. Studies demonstrating novel applications of nanoparticles were also featured prominently in our recent publications, including the use of nanoparticles for improved residence of sunscreens on the skin^18^ and siRNA delivery for applications in lymphoma and wound healing.^19^, ^20^ Papers on medical devices included control algorithms for artificial pancreas.^21^ Along with original research articles, we also published reviews on some of the active current topics in translational medicine including ionic liquids for biomedical applications,^22^ clinical landscape of nanomedicine,^23^ and translational status of nanocrystals.^24^ Reviews in *BioTM* are particularly focused on reporting translational advances and hurdles in the field.

We are thankful for the strong support from our editorial board members. In 2020, we added six new editorial board members: Jennifer West (Duke, University), Xiaoyuan Chen (NIH), David Mooney (Harvard University), Molly Stevens (Imperial College, London), Maria Jose Alonso (University of Santiago de Compostela, Spain), and Andreas Lendlein (Helmholtz Institute of Biomaterial Science, Germany). We look to our board for guidance and vision. We are especially thankful to the board members for routinely publishing their own research in *BioTM*. We feature one board member in each issue. You can read these biographies at https://aiche.onlinelibrary.wiley.com/journal/hub/journal/23806761/homepage/editorialboardprofiles.html.

We are pleased with the enthusiastic participation of the young generation of researchers in the launch and growth of *BioTM*. The support came in various forms including guest editing of special issues, submission of manuscripts, and reviewing manuscripts. Our special thanks to Prof. Aaron Anselmo of University of North Carolina, Chapel Hill, who started our feature *BioTM* Buzz, which highlights select papers from each issue. Follow BioTM_Buzz on Twitter for updates on our publications.

*BioTM* has published several special issues dedicated to several topics, including nanoparticles in medicine, nucleic acid delivery, responsive materials, and bioengineered therapeutics as well as special issues dedicated to conferences in bioengineering and translational medicine and Engineering Conferences International (ECI) nanotechnology in medicine. We also published a special issue honoring two luminaries of the field: Robert Langer and Nicholas Peppas. These special issues were edited by our enthusiastic patrons, including Profs. Kaushal Rege, Kannan Rangaramajunam, Paolo Decuzzi, Zhen Gu, Kathryn Whitehead, Pankaj Karande, Josue Snitzman, and Millicent Sullivan. We are deeply thankful for their dedication. Without the enthusiastic participation of these committed individuals, we would not have gotten off to such a great start. We also thank Arthur Baulch for excellent management of the operation, Cynthia Mascone for leadership in publication policies, and the entire Wiley team for publication strategies.

Having finished the 5‐year journey, I look forward to the next phase of the journal, which will include an increase in the number of publications each year, expansion of covered topics, and increased representation of industry and international authors in the journal. Thank you all for your strong support to the journal and look forward to your continued support in coming years.

### PEER REVIEW

The peer review history for this article is available at https://publons.com/publon/10.1002/btm2.10178.
